# Genetic population structure of *Anopheles gambiae *in Equatorial Guinea

**DOI:** 10.1186/1475-2875-6-137

**Published:** 2007-10-15

**Authors:** Marta Moreno, Patricia Salgueiro, José Luis Vicente, Jorge Cano, Pedro J Berzosa, Aida de Lucio, Frederic Simard, Adalgisa Caccone, Virgilio E Do Rosario, João Pinto, Agustín Benito

**Affiliations:** 1 Centro Nacional de Medicina Tropical. Instituto de Salud Carlos III. C/Sinesio Delgado 4, 28029 Madrid, Spain; 2 Centro de Malária e outras Doenças Tropicais, Instituto de Higiene e Medicina Tropical, Universidade Nova de Lisboa, Lisbon, Portugal; 3 Centro de Referencia para el Control de Endemias. Centro Nacional de Medicina Tropical, Instituto de Salud Carlos III, Bata, Equatorial Guinea; 4 Institut de Recherche pour le Développement, Unité 016, Montpellier, France; 5 Organisation de Coordination pour la Lutte contre les Endémies en Afrique Centrale, Yaoundé, Cameroun; 6 Yale Institute for Biospheric Studies and Department of Ecology and Evolutionary Biology, Yale University, New Haven, USA

## Abstract

**Background:**

Patterns of genetic structure among mosquito vector populations in islands have received particular attention as these are considered potentially suitable sites for experimental trials on transgenic-based malaria control strategies. In this study, levels of genetic differentiation have been estimated between populations of *Anopheles gambiae *s.s. from the islands of Bioko and Annobón, and from continental Equatorial Guinea (EG) and Gabon.

**Methods:**

Genotyping of 11 microsatellite loci located in chromosome 3 was performed in three island samples (two in Bioko and one in Annobón) and three mainland samples (two in EG and one in Gabon). Four samples belonged to the M molecular form and two to the S-form. Microsatellite data was used to estimate genetic diversity parameters, perform demographic equilibrium tests and analyse population differentiation.

**Results:**

High levels of genetic differentiation were found between the more geographically remote island of Annobón and the continent, contrasting with the shallow differentiation between Bioko island, closest to mainland, and continental localities. In Bioko, differentiation between M and S forms was higher than that observed between island and mainland samples of the same molecular form.

**Conclusion:**

The observed patterns of population structure seem to be governed by the presence of both physical (the ocean) and biological (the M-S form discontinuity) barriers to gene flow. The significant degree of genetic isolation between M and S forms detected by microsatellite loci located outside the "genomic islands" of speciation identified in *A. gambiae *s.s. further supports the hypothesis of on-going incipient speciation within this species. The implications of these findings regarding vector control strategies are discussed.

## Background

Malaria is an infectious disease that causes between 300–500 million annual clinical cases and 1.5–3 million deaths per year, mainly in children under five years old in sub-Saharan Africa [1]. Classical strategies of vector control developed in endemic areas of Africa, such as impregnated bed nets or indoor residual spraying, have not been as effective as expected, and malaria incidence is increasing. Among the factors involved in this failure are the lack of sustainability of vector control programmes and the emergence of insecticide resistance in mosquitoes [2].

Genetically based methods have been proposed for malaria vector control. These methods focus mainly in altering vectorial capacity through the genetic transformation of natural vector populations by means of introducing refractoriness genes or by sterile insect technologies [3]. Knowledge of the genetic structure of vector species is, therefore, an essential requirement as it should contribute not only to predict the spread of genes of interest, such as insecticide resistance or refractory genes, but also to identify heterogeneities in disease transmission due to distinct vector populations [4]. The most effective Afrotropical malaria vectors belong to the *Anopheles gambiae *complex, that comprises seven sibling species. Within the complex, *A. gambiae *sensu stricto (s.s.) is the most synanthropic species and shows remarkable genetic heterogeneity [5,6]. Cytogenetic analysis has revealed different chromosomal arrangements associated with paracentric inversions [5]. This has lead to the description of five chromosomal forms based in differences in the frequencies of polymorphic arrangements, geographical distribution and ecological data [5,7]. Furthermore, analysis of the X-linked ribosomal DNA cluster suggested further genetic subdivision within *A. gambiae *s.s. and led to the description of two molecular forms, provisionally named M and S, defined based on sequence differences in transcribed and non-transcribed rDNA spacers (IGS and ITS) [8,9]. Although the offspring between M and S forms are viable and fertile [10], M-S hybrids or cross-mating between the two forms are rarely observed in nature [6,11]. Genetic differentiation between molecular forms in this primary vector is of paramount relevance for the implementation and monitoring of its control, as illustrated by the extreme differences found in the distribution of knockdown resistance mutations among sympatric M and S form populations [12,13].

Previous population genetic studies pointed to a shallow population structure within major malaria vectors throughout the African continent, possibly as a result of recent population expansion leading to substantial retention of ancestral polymorphism [14,15]. The few cases of significant population differentiation have been attributed to barriers to gene flow, either physical or biological in the case of the M-S form partitioning in *A. gambiae *s.s. [16-19] However, recent studies suggest further subdivision within each of the molecular forms, as evidenced by significant levels of genetic differentiation among populations of different chromosomal forms, revealed by microsatellites and AFLP markers [20,21].

In Equatorial Guinea, malaria is one of the main causes of morbidity and mortality, being transmitted mainly by vectors of the *A. gambiae *complex [22]. In the island of Bioko, as well as in mainland Equatorial Guinea, both M and S forms are known to occur in sympatry. Different vector control measures are being implemented, including insecticide treated bed nets and indoors residual spraying [23]. However, studies regarding the genetic structure of *A. gambiae *s.s. remain scarce for Equatorial Guinea. The geography of the country, formed by both insular and continental regions, is likely to promote a greater biological heterogeneity among its vector populations. This may have important implications for the design and implementation of nationwide malaria vector control programmes. In addition, islands are regarded as potential sites for experimental releases of transgenic mosquitoes for malaria control, increasing the need for further genetic studies of its populations [18,24].

In this study, microsatellite markers have been used to estimate levels of genetic differentiation between populations of *A. gambiae *s.s. from the islands of Bioko and Annobón and from continental localities of Equatorial Guinea and Gabon, in order to determine the extent of population substructuring and its association with barriers to gene flow.

## Methods

### Mosquito collections and species identification

**Figure 1:**
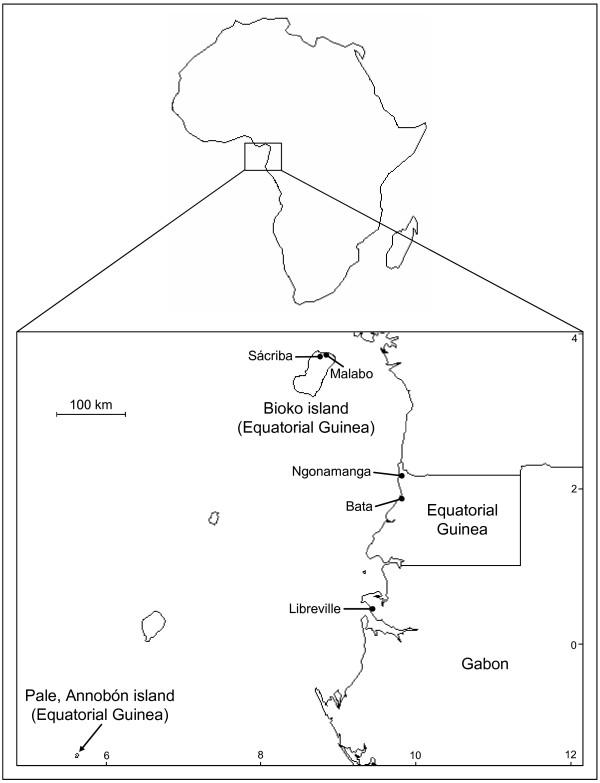
Collection sites in Equatorial Guinea and Gabon.

Entomological surveys took place in five localities of Equatorial Guinea, situated in the Gulf of Guinea, West Africa (Figure 1). In the island of Bioko, situated *ca*. 200 km from mainland Equatorial Guinea, landing and indoors resting collections were conducted in 2003 in Malabo (3°45'N/8°46'E), capital of the country, and in the village of Sácriba (3°42'N/8°43'E) 9 km away. On the island of Annobón, located in the South hemisphere 670 km away from Bioko and 585 km off mainland Equatorial Guinea, samples were collected by CDC light traps and landing catches in 2004. In the mainland, collections were carried out in 2004 in Bata (1°52'N/9°46'E) and Ngonamanga (2°08'N/9°46'E) 30 km apart, by the same sampling methods. Climatic and ecological data from these sites have been described elsewhere [22].

Mosquitoes were morphologically identified using the identification keys of Gillies & Coetzee [25]. Specimens were kept individually in silica gel filled tubes at 4°C, until DNA extraction was performed according to Collins *et al *[26]. Species identification within the *A. gambiae *complex was done by PCR according to Scott *et al *[27]. *Anopheles gambiae *s.s. molecular forms were determined as described in Favia *et al *[28]. Although cytological analysis was not performed, the Forest cytoform of *A. gambiae *s.s. is likely to be the only one present in these localities [5,22].

An additional sample from Libreville (0°23'N/9°27'E), Gabon, was also included in the analysis. This sample was collected in 2000 and it is composed by S-form *A. gambiae *s.s. [29].

### Microsatellite analysis

Eleven microsatellite loci [17,30] were genotyped: Ag3H128, Ag3H249, Ag3H119, Ag3H242, Ag3H577, Ag3H555, Ag3H59, Ag3H758, Ag3H88, Ag3H93 and 45C1. Only loci of chromosome 3 were used to avoid possible bias due to selective effects associated with paracentric inversions or reproductive isolation putative regions that are known to occur in chromosomes 2 and X [31,32]. Each locus was amplified by PCR using fluorescently labelled (FAM, NED, or HEX) forward primers [33]. Amplified fragments were separated by capillary electrophoresis in an automatic sequencer (ABI 3730, Applied Biosystems) and sizes scored using the software GeneMarker (SoftGenetics, USA).

### Data analysis

Genetic diversity by locus and sample was characterized by estimates of unbiased expected heterozygosity (*H*_*e*_, [34]), and allele richness [35], available in FSTAT v 2.9.3.2 [36]. The latter estimate was used instead of the number of alleles per locus to account for differences in sample sizes. To account for differences in sample size, these estimates were re-calculated using randomly selected sub-samples of each locality of size equal to the smallest sample size. Genotypic frequencies were tested against Hardy-Weinberg Equilibrium (HWE) proportions by exact probability tests performed in GENEPOP v.3.4 [37]. Linkage disequilibrium to confirm independence between loci was tested by exact tests on contingency tables, also available in GENEPOP.

Heterozygosity tests [38] were used to detect deviations from mutation-drift equilibrium (MDE). These tests compare two estimates of expected heterozygosity, one based on allele frequencies (*H*_*e*_), assuming Hardy-Weinberg proportions, and another based on the number of alleles and sample size (*H*_*eq*_), assuming MDE. At MDE, both estimates should be similar in the majority of loci analysed (*i.e. H*_*e*_*=H*_*eq*_). If a population experiences a bottleneck, rare alleles will be rapidly lost and therefore *H*_*eq *_will decrease faster than *H*_*e *_(*i.e. H*_*e *_> *H*_*eq*_). This apparent excess of heterozygosity in a significant number of loci is an indicator of a bottleneck, whereas the converse (*i.e. H*_*e *_<*H*_*eq*_) may indicate a population expansion. Estimates of expected heterozygosity under MDE were calculated under the Stepwise Mutation Model (SMM) and Two Phase Models (TPM) with 10–30% indels larger than the repeat unit. Calculations were done using the software BOTTLENECK 1.2.02. [38].

Differentiation among populations was measured by the fixation index *F*_ST_, calculated according to Weir and Cockerham [39] using ARLEQUIN v.3.01 [40]. Permutation tests (10,100 permutations) were performed in order to determine if estimates differed significantly from zero. The correlation between genetic and geographical distances, assuming isolation by distance, was assessed by the regression *F*_ST_/(1-*F*_ST_) on the logarithm (ln) of pairwise geographical distances [41]. Significance of the correlation coefficient was tested using Mantel tests available in GENEPOP.

To compute the probability that an individual belonged to each reference population, assignment tests were performed on the basis of multilocus genotype data using GENECLASS 2.0 [42]. The Bayesian method of Rannala and Mountain [43] was used as the computation criterion and a re-sampling algorithm based on Paetkau *et al *[44] was employed. Data was run using 10,000 simulations and a threshold of significance α = 0.01.

Finally, a Bayesian approach was used to infer the number of clusters (*K*) in the data set without prior information of the sampling locations, available in STRUCTURE 2 [45]. A model where the allele frequencies were correlated within populations was assumed (λ was set at 1, the default value). The software was run with the option of admixture, allowing for some mixed ancestry within individuals, and α was allowed to vary. Twenty independent runs were done for each value of *K *(K = 1 to 9), with a burn-in period of 100,000 iterations and 100,000 replications. The method of Evanno *et al *[46] was used to determine the most likely number of clusters. This approach uses an *ad hoc *quantity, Δ*K*, based on the second order rate of change of the likelihood function between successive values of *K*.

Whenever multiple tests were performed the nominal significance level (α = 0.05) was adjusted by the sequential Bonferroni procedure [47].

## Results

### Species and molecular form identification

A total of 213 female *A. gambiae *s.s. were analysed in this study. Of these, 133 individuals were of the M molecular form, corresponding to the samples of Ngonamanga (45) and Bata (28) on the continent, Malabo (36) in the island of Bioko and Annobón (24). The sample of Sácriba (35), in Bioko, and the sample of Gabon (45) were composed by S-form individuals. Both molecular forms were found in sympatry in Ngonamanga and in both localities of Bioko island. However, the low numbers (*N <*20) of S-form individuals collected in these localities (or M-form in the case of Sácriba) precluded further analyses. The samples of Annobón and Bata had only M-form individuals and in Gabon only the S-form has been reported [29].

### Within population genetic variability

Polymorphism at microsatellite loci varied, with allelic richness per locus ranging between four (Ag3H577 and 45C1) and 11 (Ag3H128). Two loci, Ag3H555 and 45C1, were monomorphic in Annobón. This island showed the lowest average allelic richness (3) compared to all other localities (7–8) and also had the lowest mean expected heterozygosity (0.436). The lowest genetic diversity cannot be explained by the low sample size for Annobón, as comparable differences were obtained when data was re-analysed using randomly selected sub-samples of *N *= 24 for all sites other than Annobón (Table 1). The average expected heterozygosity across all samples ranged from 0.540 (Ag3H577) to 0.789 (AgH128), with significant heterozygote deficits detected in four loci. Within each sample, significant heterozygote deficits were detected only in four occasions, in locus Ag3H88 (Malabo and Libreville), Ag3H758 (Ngonamanga) and Ag3H93 (Sácriba) (Table 1). Linkage disequilibrium tests revealed a single significant association, for the pair Ag3H128/Ag3H758 in Libreville. Altogether these results indicate that each sample represents a single panmictic gene pool.

**Table 1:** Genetic variability at microsatellite loci in *A. gambiae *s.s. from the localities surveyed

Locus		Annobón [M] Far-island (24)	Malabo [M] Near-island (36)	Sácriba [S] Near-island (35)	Bata [M] Mainland (28)	Ngonamanga [M] Mainland (45)	Libreville [S] Mainland (45)	All Samples (213)
Ag3H242	*R*_*s*_	3	5	7	4	6	5	5
	*H*_*e*_	0.613	0.654	0.635	0.634	0.679	0.646	0.644
Ag3H128	*R*_*s*_	4	10	10	16	17	7	11
	*H*_*e*_	0.659	0.849	0.721	0.919	0.921	0.666	0.789
Ag3H249	*R*_*s*_	5	6	8	8	7	7	7
	*H*_*e*_	0.702	0.811	0.789	0.835	0.795	0.792	0.787
Ag3H119	*R*_*s*_	2	6	10	5	7	9	7
	*H*_*e*_	0.190	0.688	0.839	0.745	0.738	0.826	0.671
Ag3H555	*R*_*s*_	1	6	7	7	5	6	5
	*H*_*e*_	-	0.763	0.748	0.738	0.614	0.785	**0.608**
Ag3H577	*R*_*s*_	2	5	5	4	5	5	4
	*H*_*e*_	0.386	0.569	0.626	0.536	0.611	0.509	0.540
Ag3H59	*R*_*s*_	4	7	10	7	7	7	7
	*H*_*e*_	0.727	0.652	0.873	0.705	0.763	0.746	0.744
Ag3H88	*R*_*s*_	3	10	7	10	10	6	8
	*H*_*e*_	0.519	**0.840**	0.801	0.838	0.871	**0.731**	**0.767**
Ag3H758	*R*_*s*_	2	11	9	12	13	7	9
	*H*_*e*_	0.500	0.882	0.787	0.886	**0.896**	0.669	**0.770**
Ag3H93	*R*_*s*_	2	5	7	7	7	12	7
	*H*_*e*_	0.504	0.757	**0.675**	0.731	0.718	0.862	**0.708**
45C1	*R*_*s*_	1	5	5	5	4	5	4
	*H*_*e*_	-	0.701	0.651	0.722	0.694	0.593	0.560
All loci	*R*_*s*_	3	7	8	8	8	7	7
	*H*_*e*_	0.436	**0.742**	**0.740**	**0.754**	**0.755**	0.711	**0.690**
*N *= 24	*R*_*s*_	3	8	7	8	7	7	9
	*H*_*e*_	0.436	**0.768**	**0.744**	**0.763**	0.740	0.706	**0.693**

First row indicates collection sites and location (island or mainland), molecular form in square brackets [M or S] and sample size in parenthesis; *R*_*s*_: Allele richness; *H*_*e*_: Nei's unbiased estimate of expected heterozygosity. All loci/samples: mean values over loci or populations; In bold: significant heterozygote deficits according to exact tests against Hardy-Weinberg proportions after corrections for multiple testing by the sequential Bonferroni procedure. *N *= 24: estimates made with a sample size of 24 individuals for all localities.

Cornuet and Luikart's [38] heterozygosity tests showed significant deviations from MDE in the Annobón sample under all mutational models, with an apparent heterozygote excess indicating a recent bottleneck (Table 2). In the Malabo sample, all tests were non-significant regardless of the mutation model. For the remaining samples, a significant number of loci showing an apparent heterozygote deficit were detected at least under the SMM, suggesting recent population expansion.

**Table 2:** Cornuet and Luikart's heterozygosity tests in *A. gambiae *s.s. from Equatorial Guinea and Gabon

		SMM	TPM (90%)	TPM (80%)	TPM (70%)
Annobón [M] (far-island)	*H*_*e *_> *H*_*eq*_	8	8	8	8
	*P *_(*He *> *Heq*)_	**0.005**	**0.003**	**0.002**	**0.002**
Malabo [M] (near-island)	*H*_*e *_> *H*_*eq*_	3	6	7	8
	*P *_(*He *> *Heq*)_	0.913	0.517	0.289	0.160
Sácriba [S] (near-island)	*H*_*e *_> *H*_*eq*_	1*	2*	3	4
	*P *_(*He *> *Heq*)_	1.000	0.998	0.991	0.926
Bata [M] (mainland)	*H*_*e *_> *H*_*eq*_	4*	4	4	5
	*P *_(*He *> *Heq*)_	0.992	0.794	0.585	0.382
Ngonamanga [M] (mainland)	*H*_*e *_> *H*_*eq*_	2*	2	5	5
	*P *_(*He *> *Heq*)_	0.998	0.966	0.768	0.740
Libreville [S] (mainland)	*H*_*e *_> *H*_*eq*_	1*	2*	4	5
	*P *_(*He *> *Heq*)_	1.000	0.995	0.912	0.768

First column indicates collection sites and location (island or mainland), and molecular form in square brackets [M or S]. SMM: stepwise mutation model; TPM: two-phase mutation model with indels larger than one repeat of 10%, 20% and 30%, respectively; *H*_*e *_> *H*_*eq*_: number of loci showing a heterozygote excess (11 polymorphic loci in all localities except Annobón with 9); *P*_(*He *> *Heq*)_: *P-value *of Wilcoxon tests to determine the significance of the number of loci in which *H*_*e *_> *H*_*eq*_. In bold are *P-values *that remained significant after adjustment by the sequential Bonferroni procedure. *: Significant number of loci in which *H*_*e *_<*H*_*eq *_by Wilcoxon tests, also after adjustment of the nominal significance value.

### Genetic differentiation and isolation by distance

Pairwise estimates of *F*_ST _over all loci between samples are presented in Table 3. The Annobón sample showed the highest degree of differentiation from all other samples (*F*_ST _= 0.196–0.269, *P *< 0.001). Genetic differentiation was higher between M and S form samples within Bioko island (*F*_ST _= 0.089, *P *< 0.001) than between the M-form samples from Bioko and mainland Equatorial Guinea (*F*_ST _= 0.023–0.042, *P *< 0.001) or between the S-form samples from Bioko and Gabon (*F*_ST _= 0.050, *P *< 0.001). The only non-significant *F*_ST _estimate was the one involving the two continental M-form samples, Bata and Ngonamanga. No statistically significant correlations were detected between genetic differentiation, measured by *F*_ST_, and geographic distances (Mantel tests: r = 0.62, *P *= 0.051), even when tests were carried out with M-form samples only (Mantel tests: r = 0.84, *P *= 0.084).

**Table 3:** Estimates of pairwise *F*_*ST *_among populations of *A. gambiae *s.s. from Equatorial Guinea and Gabon.

	Annobón	Malabo	Sácriba	Bata	Ngonamanga
Annobón [M] (far-island)	-				
Malabo [M] (near-island)	**0.212**	-			
Sácriba [S] (near-island)	**0.269**	**0.089**	-		
Bata [M] (mainland)	**0.196**	**0.023**	**0.080**	-	
Ngonamanga [M] (mainland)	**0.187**	**0.042**	**0.079**	0.003	-
Libreville [S] (mainland)	**0.267**	**0.109**	**0.050**	**0.100**	**0.099**

First column indicates collection sites and location (island or mainland), and molecular form in square brackets [M or S]. In Bold: significant estimate according to permutation tests (10,100 permutations)

Results of the assignment tests [42] showed that on average 62.9% (134 out of 213) of the individuals were correctly assigned to their original sampling site (Table 4). The collections from Annobón and Sácriba presented the highest proportion of correctly assigned individuals (0.88 and 0.89, respectively). Over 81% of the 79 mis-assignments occurred between samples of the same molecular form, independent of geographic origin (within M-form: 0.62; within S-form: 0.19; between forms: 0.19). Within the M-form, nearly half of the individuals from Malabo, in Bioko island, were assigned to mainland Bata and nearly 60% of individuals from Ngonamanga were also mis-assigned to Bata. Similarly, within the S-form over 30% of the individuals from Libreville were mis-assigned to Sácriba, in Bioko.

**Table 4:** Results of assignment tests based on microsatellite gene frequencies among samples of *A. gambiae *s.s. from Equatorial Guinea and Gabon.

	Annobón [M]	Malabo [M]	Sácriba [S]	Bata [M]	Ngonamanga [M]	Libreville [S]
Annobón [M] (far-island)	**0.88**	**-**	**-**	0.12	**-**	**-**
Malabo [M] (near-island)	**-**	**0.44**	0.08	0.42	0.06	**-**
Sácriba [S] (near-island)	**-**	**-**	**0.89**	0.03	0.05	0.03
Bata [M] (mainland)	**-**	**-**	0.07	**0.82**	0.11	**-**
Ngonamanga [M] (mainland)	**-**	**-**	0.16	0.57	**0.27**	**-**
Libreville [S] (mainland)	**-**	**-**	0.31	**-**	**-**	**0.69**

**Figure 2:**
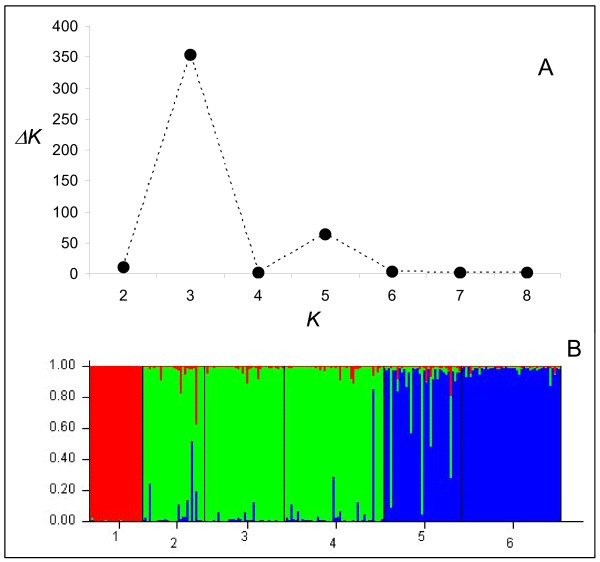
Bayesian cluster analysis using STRUCTURE. A: estimates of ΔK, based on the second order rate of change of the likelihood function with respect to *K*, to determine the most likely number of clusters (*K*) in the data set. In this case *K *= 3. B: graphical representation of the data set for the most likely *K *= 3, where each colour corresponds to a suggested cluster and each individual is represented by a vertical bar. The numbers in the X-axis correspond to a specific sample: 1- Annobón, 2- Bata, 3- Malabo, 4- Ngonamanga, 5- Sácriba and 6- Libreville. The Y-axis represents the probability of assignment of an individual to each cluster.

First column indicates collection sites and location (island or mainland), and molecular form in square brackets [M or S]. Values are proportions of individuals from the original sample (lines) assigned to each locality (columns). Proportions of individuals correctly assigned are shown in bold;

Bayesian cluster analysis performed with STRUCTURE [45] showed that the most likely *K *value identified was *K *= 3 (Figure 2a). This corresponds to three distinct genetic clusters (Figure 2b): (1) M-form *A. gambiae *s.s. from Annobón Island; (2) M-form samples from Bioko island (Malabo) and the mainland (Bata and Ngonamanga); (3) S-form samples from Bioko island (Sácriba) and the mainland (Libreville).

## Discussion

Genetic diversity varied among island *A. gambiae *s.s.populations from Equatorial Guinea. In Bioko, levels of genetic variation in both M and S-forms were quite similar to those observed in the three continental samples. When compared with neighbouring mainland countries, mean expected heterozygosity values were similar to those reported for both molecular forms in Cameroon (*H*_*e*_: 0.77–0.81; [16]), Nigeria (*H*_*e*_: 0.76–0.79; [48]) and Ghana (*H*_*e*_: 0.71–0.82; [49]). In other islands in close proximity with mainland, genetic diversity was also similar to that of adjacent continental ones [24]. In contrast, the sample from the island of Annobón showed much lower levels of genetic diversity than Bioko samples. Bioko and Annobón lie at the opposite extremes of a volcanic chain in the Gulf of Guinea, which also includes the archipelago of São Tomé and Príncipe (STP). In these islands, estimates of genetic diversity were intermediate to those found in Bioko and Annobón (*H*_*e*_: 0.45–0.55; [18]). These findings overall agree with principles of island biogeography, in which biological (and genetic) diversity is positively correlated with the size of the island and negatively correlated with distance from mainland [50].

The heterozygosity tests suggested an expansion process in both M and S-form continental populations of *A. gambiae *s.s., in agreement with previous works based on mainland populations of this species [51]. Within the island of Bioko, the differences found between M and S samples may indicate different historical processes. While the M-form was found at MDE, the S-form appears to be expanding. This pattern could be due to different timings of arrival of the two molecular forms on the island. The M-form *A. gambiae *s.s. individuals might have been the first to be introduced and thus have reached MDE, while the genetic signature of population expansion in the S-form sample could be due to its more recent establishment on the island.

The signature of population contraction detected in Annobón may be a consequence of several factors that need further examination. These may include a more recent colonization of this island, periodical strong fluctuations in effective population size, or a synergistic combination of these factors. Historical data indicates that whilst the first humans arrived to Bioko island *ca*. 3,000 years ago, Annobón was colonized only in the 15^th ^century by slaves from Angola and São Tomé and Príncipe [52]. Periodic demographic oscillations occur in Annobón, associated with seasonal migrations of workers to Bioko island. In addition, most of the human population from the main village (Pale) moves to smaller inland villages during the hurricane season. These demographic fluctuations in the human population might lead to periodical changes in the effective size of the local mosquito populations.

Significant genetic differentiation was observed between M and S-form samples that are less than 10 km apart within Bioko island. The estimate of *F*_*ST *_was 2 to 4-fold greater than those among M-form samples from Bioko and the continent (210–236 km apart) and nearly 2-fold greater than the one obtained between the S-form samples of Bioko and Libreville (378 km apart). Similarly, higher *F*_*ST *_values were obtained in all comparisons between the S-form from Libreville and M-form samples from mainland or Bioko island, when compared to the estimate between island and continental S-form. It is unlikely that *F*_*ST *_estimates may have been influenced by the different time of collections between the sample of Libreville (2000) and those from Equatorial Guinea (2003–2004). Microsatellite allele frequencies in *A. gambiae *s.l. tend to vary little over generations, reflecting large effective population sizes [53,54]. The higher differentiation between M and S forms was also evident from the assignment tests performed in the present study, in which most mis-assignments were shared between samples of the same molecular form regardless of its geographic origin. Bayesian cluster analysis further supported this partitioning, by grouping together M and S form samples in two separate clusters, again independently of sample location. In a previous study, a significant *F*_*ST *_estimate (0.070) had also been obtained by microsatellite analysis, between sympatric M and S forms from Malabo [55].

Altogether, these results agree with the notion of a biological discontinuity within *A. gambiae *s.s., and that M and S forms are likely to be the result of an on-going incipient speciation process [6]. Evidence of limited gene flow between molecular forms has been described in other West African countries, with different genetic markers [4,8,13,56,57]. However, several studies, some of which based on microsatellites, suggest that the highest genetic differentiation between M and S forms appears to be restricted mainly to certain genomic regions, particularly in the low-recombination centromeric regions of chromosome X and chromosome 2L [32,58-61]. This led the authors to hypothesise that these regions contain genes responsible for reproductive isolation. In this study, high differentiation between M and S-forms was detected by the analysis of microsatellites mapped in chromosome 3, *i.e*. outside regions where putative isolation genes are thought to occur, reinforcing the idea of high levels of genetic isolation between molecular forms in this geographic region. Similarly, Wondji *et al *[16] also observed high differentiation between sympatric M and S-forms in Cameroon, with the analysis of microsatellites located outside the centromeric regions of chromosomes 2L and X. Whilst their results may appear conflicting with those from Turner *et al *[59], given that both studies were based on samples from Cameroon, this may not be case as different genetic markers (*i.e*. microsatellites and microarray probes) were used. Microsatellites detect allele frequency differences in highly polymorphic regions of the genome, while hybridization approaches using microarrays will detect differentiation in regions where polymorphism is relatively low within each form relative to differences between forms, such as the case of centromeric regions. On the other hand, in a recent microsatellite-based study carried out in Ghana, levels of population differentiation in *A. gambiae *s.s.were more attributable to ecological zones rather than to the M-S molecular form partitioning [49]. These apparent differences may suggest that, although it is clear that incipient speciation is on-going within *A. gambiae *s.s., the degree of isolation between its reproductive units is likely to vary throughout the species eco-geographic distribution range.

Within the M-form, the low levels of differentiation between the sample of Bioko and those from continental Equatorial Guinea suggest that gene flow between this island and the mainland is likely to occur. Reimer *et al *[55] detected slightly higher levels of population differentiation between Bioko island (Malabo) and sites from the nearest continental country, Cameroon (*F*_*ST*_: 0.038–0.057). Being the capital of the country, connections with continental Equatorial Guinea (Bata), by air or sea at a daily frequency, may promote gene flow through human-mediated transportation of mosquitoes. Several studies provided evidence of human activities promoting gene flow in mosquito populations between islands or between islands and mainland [62]. Conversely, the highest levels of population differentiation were found in all comparisons that involved the M-form sample of Annobón island. This supports a higher degree of isolation of this island and agrees with previous studies demonstrating the ocean and other extensive water-bodies as a physical barrier to gene flow in anopheline species [18,19,24,63]. Similarly, microsatellite-based studies conducted in the neighbouring STP islands also showed high levels of differentiation with the continent (*F*_*ST*_: 0.118–0.250) [18] and subsequent sequencing analysis of rDNA and mitochondrial DNA regions suggests only two main colonization events of *A. gambiae *s.s. into these islands [Marshal *et al*, unpublished].

## Conclusion

In the present study, strong levels of population substructure were detected in *A. gambiae *s.s. from Equatorial Guinea. Patterns of genetic differentiation are most likely governed by the presence of both physical/geographic (the ocean) and biological (the M-S form discontinuity) barriers to gene flow. These findings have important practical implications for the management of vector control strategies. The biological partitioning between M and S-forms may influence the evolution of genes of interest such as insecticide resistance genes. An unusual frequency of knockdown resistance (*kdr*) mutations has been detected in the M-form population of Bioko, contrasting with the absence of these alleles in the S-form of this island [55]. This implies that a detailed characterization of the distribution of M and S forms at a local level and continuous monitoring of *kdr *mutations within each form would be desirable for a rational management of insecticides for malaria control. The closest proximity and lowest differentiation with mainland coupled with the genetic isolation found between sympatric M and S form populations in Bioko, could make this island inappropriate for initial experimental releases of genetically modified mosquitoes, as only part of the vector population might be affected. On the other hand, in Annobón the presence of a single molecular form coupled with its higher geographic and genetic isolation, might render this island comparatively more suitable for transgenic-based malaria control.

## Authors' contributions

MM was involved in the design of the survey, microsatellite genotyping, data analysis and manuscript preparation. PS participated in data analysis and drafting the manuscript. JLV carried out microsatellite and data analysis. JC participated in field surveys and helped drafting the manuscript. PB and AL participated in molecular analyses and in the elaboration of the manuscript. FS and AC were involved in sample collections, molecular analyses and revised the manuscript. VER participated in the design of the study and revised the manuscript. JP conceived and co-supervised the study, assisted data analysis and coordinated the draft of the manuscript. AB participated in the conception and design of the study, revised the manuscript and provided overall supervision to the work. All authors read and approved the final manuscript.
